# Epidemiological Trends of Drug-Resistant Tuberculosis in China From 2007 to 2014: A Retrospective Study

**DOI:** 10.1097/MD.0000000000003336

**Published:** 2016-04-18

**Authors:** Xiao-chun He, Xian-xin Zhang, Jiang-nan Zhao, Yao Liu, Chun-bao Yu, Guo-ru Yang, Huai-chen Li

**Affiliations:** From the Department of Respiratory Medicine (X-CH, YL, H-CL), Shandong Provincial Hospital Affiliated to Shandong University, Jinan; College of Pharmacy (X-CH), Shandong University, Jinan; Department of Respiratory Medicine (X-XZ), Shandong Provincial Chest Hospital, Jinan; Department of Respiratory Medicine (J-NZ), The First Hospital of Jiaxing, Jiaxing; Katharine Hsu International Research Center of Human Infectious Diseases (C-BY), Shandong Provincial Chest Hospital, Jinan; and Department of Respiratory and Critical Care Medicine (G-RY), The Second People's Hospital of Weifang, Weifang, China.

## Abstract

The emergence and spread of drug-resistant tuberculosis (DR-TB) has become the major concern in global TB control nowadays due to its limited therapy options and high mortality. A comprehensive evaluation for the epidemiological trends of DR-TB in mainland China, of which TB incidences remain high, is essential but lacking. This study aimed to describe the trends of DR-TB overtime, especially multidrug-resistant TB (MDR-TB); and to identify unique characteristics of MDR-TB cases compared with drug-susceptible TB cases in Mainland China.

We retrospectively analyzed surveillance data collected from 36 TB prevention and control institutions in Shandong Province, China over an 8-year period. Unique characteristics of MDR-TB were identified; Chi-square test for trends and linear regression were used to assess the changes in proportions of different resistance patterns overtime.

The overall MDR rate was 6.2% in our sample population. There were no statistically significant changes in the percentage of drug-susceptible, isoniazid (INH) resistance, ethambutol (EMB) resistance, streptomycin (SM) resistance, and MDR TB during our study period except that the overall rifampin (RFP) resistance and rifampin monoresistance (RMR) increased at a yearly rate of 0.2% and 0.1%, respectively. Among those with known treatment histories, a higher MDR rate of 8.7% was observed, in which 53.9% were primary MDR-TB patients, and this rate was increasing at a yearly rate of 4.1% over our study period. MDR-TB patients were more likely to be female (odds ratio [OR], 1.23; 95% confidence interval [CI], 1.05–1.34), aged 25 to 44 years (OR, 1.67; 95%CI, 1.45–1.93), retreated (OR, 11.95; 95%CI, 9.68–14.76), having prior TB contact (OR, 1.89; 95%CI, 1.19–2.78) and having cavity (OR, 1.57; 95%CI 1.36–1.81), or bilateral disease (OR, 1.45; 95%CI 1.19–1.76) on chest radiology.

Persistent high levels of MDR-TB, increasing rates of primary MDR-TB and RMR characterize DR-TB cases in mainland China; community-acquired drug resistance may be one of the most modifiable factors in future TB control strategies.

## INTRODUCTION

Drug-resistant tuberculosis (DR-TB) has aroused global public concern afresh since the early 1990s because of major outbreaks of these organisms in developed countries.^1,2^ In 1986, rifampin (RFP) resistance was demonstrated to be a threat to the success of modern short-course treatment of TB since it increased failure during chemotherapy and relapse rate afterwards.^3^ Multidrug-resistant TB (MDR-TB), defined as TB resistant to at least isoniazid (INH) and RFP, is more intractable due to its longer, more toxic, and expensive treatment regimens as well as its poorer treatment outcomes.^4–7^ Moreover, it puts TB patients only 1 step toward extensively drug-resistant. Globally in 2013, 9 million people was infected with TB in which 480,000 (5.3%) were estimated to have MDR-TB, and 1.5 million died from the disease. There is no doubting that TB remains one of the deadliest infectious diseases in the world.^8^

China is among the 22 high TB and MDR-TB burden countries and accounts for 1/4 of the MDR-TB population in the world.^8,9^ In 2007, a national survey of DR-TB was conducted by the Chinese Center for Disease Control and Prevention, estimated that the MDR-TB rate was 8.3% among TB cases.^9^ Moreover, there were numerous reports of DR-TB prevalence from different regions across the country in recent years, showing MDR-TB rate ranging from 6.5% to 16.6%.^10–13^ Since 1990, China has made great progress in TB control and prevention, TB incidence rate was estimated to have declined by 3.4% per year. However, the high level of DR-TB rate all over the country has drawn our attention to the effectiveness of existing TB control policies toward DR-TB.

In China, defining the secular trends and unique characteristics of DR-TB can give a better understanding of current status of TB epidemic, which is scarce but essential to inform health care professionals, public health programs, and policy makers. We sought to provide them evidence-based information and thus to design a more rational approach to effective prevention and control of DR-TB, and to provide experience in monitoring longitudinal changes in places with similar settings.

We compared baseline characteristics for patients with MDR-TB and changes of different drug-resistant patterns overtime on the basis of drug susceptibility testing (DST) for first-line drugs in a cohort of patients from 36 TB prevention and control institutions in Shandong Province, China over an 8-year period.

## MATERIALS AND METHODS

### Ethics

The study was approved by the Ethic Committee of Shandong Provincial Hospital, affiliated to Shandong University and the Ethic Committee of Shandong Provincial Chest Hospital (SPCH). Patient records were anonymized and deidentified prior to analysis.

### Study Population and Data Collection

Shandong province lies in the east coast of China and is the 2nd largest province with a population of 95 million. In 2013, 35,971 people in Shandong were infected with TB. This retrospective study enrolled consecutive culture-confirmed TB cases reported in 36 TB prevention and control institutions of Shandong Province, China from January 1, 2007 to December 31, 2014. Twenty-one county-level and 13 municipal-level local health departments as well as 2 province-level hospitals: Shandong Provincial Hospital and SPCH were involved in the present study. Among which SPCH is the only provincial TB specialized hospital in Shandong province. Trained research clinicians collected and recorded the patients’ information confirmed within their jurisdiction using a standard case report form and reported them to the provincial health department – Katharine Hsu International Research Center of Human Infectious Diseases. Katharine Hsu International Research Center of Human Infectious Diseases was set up by SPCH and Center for TB Control and Prevention of Shandong province in 2004, and has been responsible for the laboratory quality assurance and TB surveillance in Shandong province.

Drug susceptible test (DST) data had been collected for the first-line drugs INH, RFP, ethambutol (EMB), and streptomycin (SM). Sociodemographic data including age, sex, occupation, alcohol use, and smoking were collected; clinical data including treatment history, prior TB contact, cavity, and bilateral disease on chest radiology were available.

### Drug Susceptibility Testing

DST was performed using the proportion method on acid-buffer Löwenstein–Jensen (L-J) medium. Specimens were digested and decontaminated with 4% sodium hydroxide for 15 minutes and then inoculated to L-J media. Susceptibility testing for INH, RFP, EMB, and SM was performed on L-J medium at the following concentrations^14^ 0.2, 40, 2.0, and 4.0 μg/mL, respectively. The isolates were considered to be resistant if there was more than 1% growth on medium containing anti-TB drugs as compared with the growth on drug-free medium. All laboratories at study sites were subjected to external quality assessment through TB National Reference Laboratory Network.

### Data Inclusion and Definitions

All the TB cases that had a positive *Mycobacterium tuberculosis* culture with DST results as well as demographic and clinical information were included. We excluded patients with nontuberculous mycobacteria. Patients infected with human immunodeficiency virus (HIV) were not included since HIV-positive patients are transferred to HIV specialized hospital immediately in China.

Drug-susceptible TB was defined as TB susceptible to both INH and RFP. MDR-TB was defined as TB with resistance to at least INH and RFP. A new TB case is defined as a patient who has never been treated for TB or has taken anti-TB drugs for less than 1 month; a previously treated TB case means that the case has received TB treatment before the current TB episode for 1 month or more.^15^ Accordingly, primary drug resistance was defined as drug resistance in a new TB case, and acquired resistance was defined as drug resistance in a previously treated TB case.

### Statistical Analysis

We calculated odds ratios (ORs) and 95% confidence intervals (CIs) for the comparisons of specific characteristics between MDR-TB and drug-susceptible TB cases using Pearson Chi-square test. Chi-square test for trends and linear regression were used to assess the changes in proportions of the different resistance patterns overtime. *P* value <0.05 was considered statistically significant. Statistical analyses were conducted with SPSS software, version 16.0.

## RESULTS

### Case Estimates

A total of 13,486 culture-confirmed TB cases with DST results as well as demographic and clinical information were collected during the 8-year study period. The mean age of these TB patients was 43.8 (mean ± SD, 43.8 ± 19.8) years, males accounted for 72.1% of these TB patients.

### Drug Resistance Patterns

Out of the 13,486 TB cases, 11,386 (84.4%) were drug-susceptible TB cases, 2855 (21.2%) patients were resistant to at least 1 first-line drug. SM had the highest rate of resistance (14.7%), followed by INH (13.4%), RFP (8.4%), and EMB (2.6%). A total of 838 (6.2%) cases met the study case definition for MDR-TB, and 204 (1.5%) cases were resistant to all first-line drugs (Table 1).

**TABLE 1:**
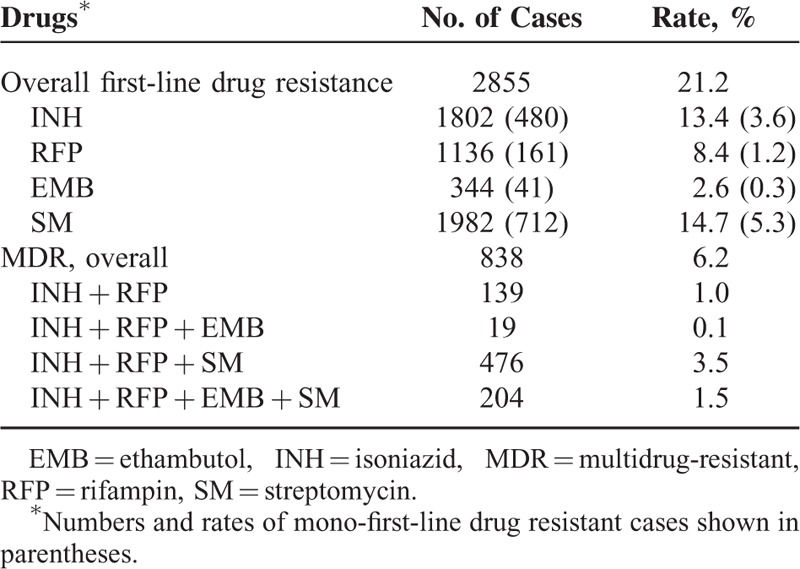
First-Line Drug Resistance of the 13,486 Tuberculosis Cases, China, 2007 to 2014

As a subset of the whole study cohort, treatment histories were available for the 5661 (41.9%) patients from SPCH, among which 4987 (88.1%) were new cases and 674 (11.9%) previously treated cases. The proportions of MDR-TB were 5.4% and 33.8% in new- and previously treated-TB cases, respectively, making an overall of 495 (8.7%) cases being identified as MDR-TB. And 267 (53.9%) patients with MDR-TB were experiencing their 1st episode of TB.

### Demographic and Clinical Characteristics

Table 2 shows the demographic and clinical characteristics for drug-susceptible and MDR-TB in detail. Compared to patients with drug-susceptible TB, patients aged 25 to 44 years were more likely to have MDR-TB (OR, 1.67; 95% CI, 1.45–1.93), while MDR-TB cases were less likely to be in cases ≥65 years (OR, 0.52; 95% CI, 0.42–0.64). Female were more likely to have MDR-TB when compared with drug-susceptible TB cases (OR, 1.23; 95% CI, 1.05–1.43). Current smokers were more frequent among patients with MDR TB (OR, 1.44; 95% CI, 1.23–1.68).

**TABLE 2:**
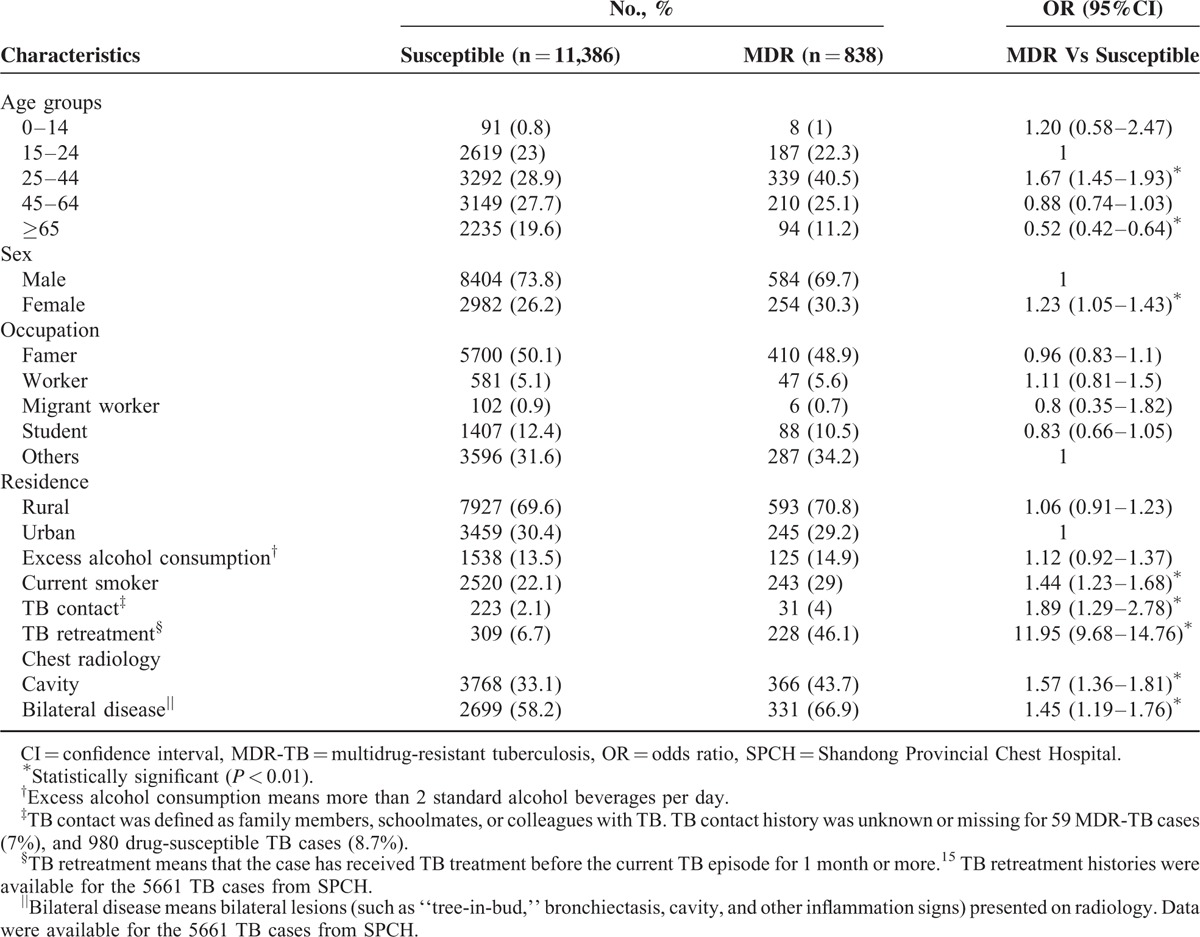
Sociodemographic and Clinical Characteristics of MDR-TB and Drug-Susceptible Tuberculosis Cases in China, 2007 to 2014

Prior TB contact rate was higher in the MDR-TB group (OR, 1.89; 95%CI, 1.19–2.78). And patients with MDR-TB were significantly more likely to have been previously treated (OR, 11.95; 95%CI 9.68–14.76). MDR-TB had a greater chance to have cavity disease (OR, 1.57; 95%CI 1.36–1.81) and to have bilateral lesions (OR, 1.45; 95%CI 1.19–1.76) on chest radiology compared to drug-susceptible TB.

### Trends Overtime

**FIGURE 1:**
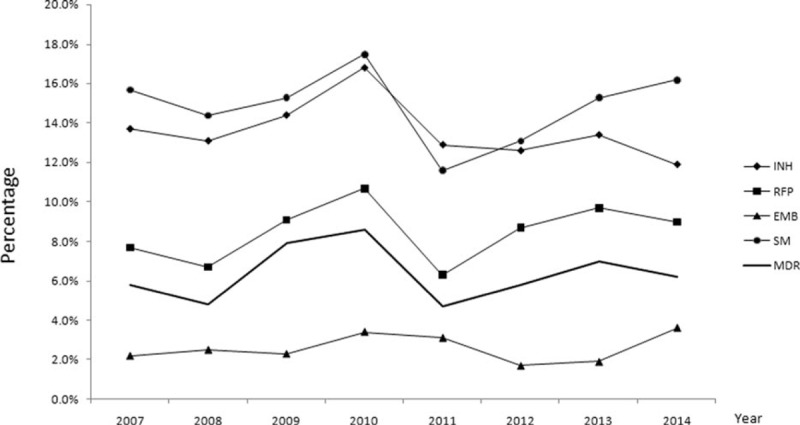
Trends of different drug-resistance patterns among 13,486 culture-confirmed TB cases in China, 2007 to 2014. For MDR-TB (χ^2^ = 0.364, *P* = 0.546); for INH resistance (χ^2^ = 3.637, *P* = 0.057); for RFP resistance (χ^2^ = 5.106, *P* = 0.024; linear regression formula: *R*^2^ = 0.13, x-coefficient = 0.002, SE = 0.075); for EMB resistance (χ^2^ = 0.494, *P* = 0.482); for SM resistance (χ^2^ = 0.045, *P* = 0.831). EMB = ethambutol, INH = isoniazid, MDR-TB = multidrug-resistant tuberculosis, RFP = rifampin, SE = standard error, SM = streptomycin, TB = tuberculosis.

For the entire study cohort (13,486 cases), the longitudinal changes in overall percentage of drug-susceptible, INH resistance, EMB resistance, SM resistance, and MDR TB overtime were not found to be statistically significant using the Chi-square test for trends (χ^2^ = 0.212, *P* = 0.645 for drug-susceptible TB; χ^2^ = 3.637, *P* = 0.057 for INH resistance; χ^2^ = 0.494, *P* = 0.482 for EMB resistance; χ^2^ = 0.045, *P* = 0.831 for SM resistance; and χ^2^ = 0.364, *P* = 0.546 for MDR-TB) and linear regression. However, we found that the overall RFP resistance was increasing at a yearly rate of 0.2% (*R*^2^ = 0.13; Chi-square test for trends: χ^2^ = 5.106, *P* = 0.024) over the 8-year period (Figure 1). Besides, monoresistance analysis showed a statistically significant increase in rifampin monoresistance (RMR) proportion (χ^2^ = 8.76, *P* = 0.003) while INH, EMB, and SM monoresistance showed no statistically significant changes.

**FIGURE 2:**
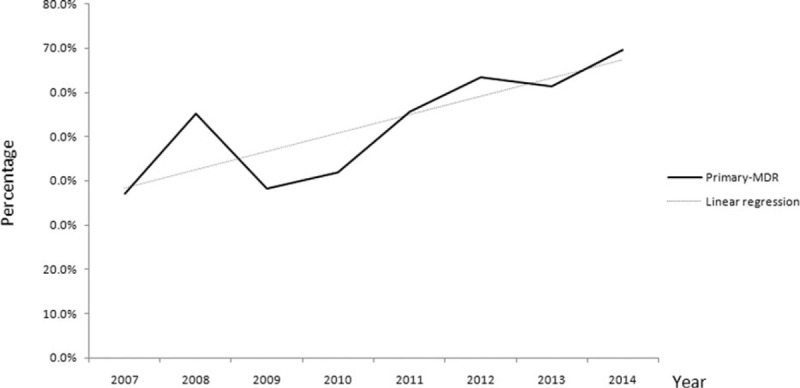
Trends of the primary MDR-TB proportion among MDR-TB cases in SPCH, 2007 to 2014. (χ^2^ = 21.555, *P* < 0.001; linear regression formula: *R*^2^ = 0.677, x-coefficient = 0.041, SE = 0.341). MDR-TB = multidrug-resistant tuberculosis, SE = standard error, SM = streptomycin, SPCH = Shandong Provincial Chest Hospital.

Treatment histories were available only for TB cases from SPCH. To have a better understanding of the epidemic trends in TB cases with different treatment histories, we analyzed this subset of patients particularly. For the 5661 patients from SPCH, we observed that the proportion of drug-susceptible TB increased from 77.1% in 2007 to 83.4% in 2014, increasing at yearly rate of 0.6% (*R*^2^ = 0.401; Chi-square test for trends: χ^2^ = 6.178, *P* = 0.013); MDR-TB declined at a yearly rate of −0.3%, but the differences did not reach statistical significance (χ^2^ = 3.521, *P* = 0.061). Further breakdown into new- and previously treated-TB cases showed that there was an increasing yearly rate of 0.1% for primary MDR-TB and a declining yearly rate of −1% for acquired-MDR-TB cases, but these changes were not statistically significant using the Chi-square test for trends (*P* = 0.135 and 0.166, respectively). It is noteworthy that the percentage of primary MDR-TB among MDR-TB patients increased from 37% in 2007 to 69.7% in 2014, increasing at a yearly rate of 4.1% (*R*^2^ = 0.677; Chi-square test for trends: χ^2^ = 21.56, *P* < 0.001) (Figure 2).

In addition, we analyzed trends of INH, RFP, EMB, and SM resistance in new- and previously treated-TB cases, respectively. We found that in new TB cases, the overall RFP resistance rate was increasing at a yearly rate of 0.2% during our study period (*R*^2^ = 0.214, Chi-square test for trends: χ^2^ = 4.342, *P* = 0.037). Although other resistance patterns in both new- and previously treated-TB cases showed no statistically significant changes overtime (in new TB cases, χ^2^ = 0.09, *P* = 0.764 for INH resistance, χ^2^ = 2.315, *P* = 0.128 for EMB resistance, and χ^2^ = 1.702, *P* = 0.192 for SM resistance; in previously treated TB cases, χ^2^ = 3.175, *P* = 0.075 for INH resistance, χ^2^ = 2.81, *P* = 0.094 for RFP resistance, χ^2^ = 0.019, *P* = 0.891 for EMB resistance, and χ^2^ = 0.418, *P* = 0.518 for SM resistance). We also analyzed the trends of primary and acquired MDR-TB in different age groups, it showed that only the increase of primary MDR-TB between 45 and 64 years was statistically significant (*P* = 0.011).

## DISCUSSION

It was a large population and long-term-based retrospective study conducted in the second largest province located on the eastern coast of China. To our knowledge, this is the 1st study providing comprehensive assessment of the burden of different DR-TB patterns from a longitudinal perspective in mainland China.

Analysis of the 13,486 culture-confirmed TB cases showed that MDR-TB was more frequent among patients aged 25 to 44 years, females, current smokers, patients with prior TB contact, patients with previous TB treatment, and patients with cavity or bilateral lesions (Table 2). There were no statistically significant changes in the percentage of different drug-resistance patterns except the increase in overall RFP resistance and RMR rate during the study period. We also found a statistically significant increase in RFP resistance rate among new TB cases and an increase in percentage of primary MDR-TB between ages 45 and 64 for the 5661 cases whose treatment histories were available.

We have noted that the 5661 TB cases from SPCH had a higher MDR-TB rate than our sample population, it can partly be explained that SPCH is a provincial TB specialized hospital with a relatively higher proportion of previously treated TB cases.^13^

In our study, we found evidence of ongoing transmission of MDR-TB strains in China. First, we documented that patients who had a prior TB contact history were more likely to have MDR-TB. There have been few reports about the relationship between prior TB contact and MDR-TB, which may possibly due to the great number of patients who were uninformed of their surrounding TB status as well as the undiagnosis or unawareness of the disease for spreaders themselves.^16^ Besides, TB contact occurs mainly in groups closely related (e.g., family members, schoolmates, and colleagues) to allow long-term exposure. Irrespective of the inaccessibility of data, prior TB contact could be a sensitive indicator for primary transmission within certain communities.^17^ In our study, though there were 7.7% of the TB contact data were unknown or missing, the large population and long-term-based DST and clinical data allowed us to have a better understanding of the relationship between prior TB contact and MDR-TB in China. The founding that TB patients who had a prior TB contact history were more likely to have MDR-TB indicated the ongoing transmission of MDR-TB in certain communities. Second, for patients whose treatment histories were available (TB cases from SPCH), more than half of the patients (53.9%) with MDR-TB were experiencing their 1st episode of TB. And the rate increased from 37% in 2007 to 69.7% in 2014, increasing at a yearly rate of 4.1% throughout the study period. The condition in our study was quite consistent with surveys conducted in countries with >700 estimated MDR-TB cases per year and in 16 European countries where primary transmission of MDR-TB strains is ongoing.^16,18^ The large proportion of MDR-TB in new TB patients was a warning sign that MDR-TB is spreading. As this proportion elevated overtime, the transmission status is getting worse. Furthermore, there was a statistically significant increase in primary MDR TB between ages 45 and 64. It merits our attention because it may have implications for designing age-group targeting policies in future TB control strategies.

It is also noteworthy that the overall RFP resistance as well as RMR rate increased over the study period. RMR was less frequent than INH monoresistance since RFP has lower occurrence of naturally occurring resistance mutations than INH does.^19^ Consequently, the emergence of RFP resistance at the end of the 20th century was thought to be a threat to the short-course treatment of TB, especially in TB cases coinfected with HIV.^20,21^ A recent study conducted in South Africa has reported a rapid increase in RMR-TB among HIV-coinfected persons,^22^ conversely, our study provided evidence of ongoing occurrence of RMR-TB free from HIV-coinfection. We speculate that the emergence and increase of this unusual pattern of drug resistance in China could be driven by various factors. First, the universal and inadequate use of rifamycin for the treatment of TB might enable selection of drug-resistant strains of RFP. Moreover, a study from New York City in 2005 demonstrated that the risk for acquired RFP resistance among HIV-infected persons with TB was depended on the thrice weekly dosing of RFP in the intensive phase of treatment.^21^ It remains to be determined if the RMR strains in China is acquired during the TB treatment with rifapentine semiweekly in persons without HIV infection. Moreover, as a previous study from the United States reported, 13% of the RMR TB cases were infected through close contact with an RMR-TB case.^20^ Our study showed an increase in overall RFP-resistance TB rate in the new TB group from a subset (TB cases from SPCH) of the sample population, but the increase of RMR-TB rate did not reach statistical significance, thus there is a possibility that primary transmission of RMR strains is ongoing. But the role that acquired RMR versus primary transmission of RMR played in the overall increase of RMR TB rate cannot be further evaluated in our patient population. INH or RMR was an independent predictor for acquired MDR-TB^9^ and therefore should be managed carefully. The treatment of RMR-TB has not been specifically addressed in the last World Health Organization guidelines,^23^ but according to the The International Union Against Tuberculosis and Lung Disease, RMR-TB should be similarly treated as MDR-TB.^24^

In line with previous studies, we identified important differences between MDR-TB cases and patients with drug-susceptible TB that may have impact on the course of disease and have implications for case management. According to our study, special attention should be paid to TB patients who are 25 to 44 years,^12,17,25–27^ previously treated,^16,28–31^ having cavity and bilateral disease on their chest radiology.

Our study is subject to the usual limitations inherent with retrospective design and data collection. First, the lack of DST for second-line drugs impeded us to further understanding of extensively drug-resistant which has already been documented in China,^9,13,32,33^ and the emergence of extremely drug-resistant and totally drug-resistant strains from other parts of the world puts TB into a functionally untreatable situation.^34–37^ To comprehensively assess these drug-resistant patterns beyond MDR, DST for second-line drugs and further corresponding studies are urgently needed. Second, TB treatment history was available only for TB patients from SPCH, thus the generalizability of this subset of data might be limited. Third, the information about education, socioeconomic status, and living conditions were not well described and recorded in the medical records, so we failed to show the relationships between these factors and the epidemic of drug-resistant TB, impeding us to further design of related strategies. Finally, since our sample population are culture-confirmed HIV-seronegative TB cases, there is an absence of correlation with the TB cases among HIV positive patients in the same population, so we failed to show if undiagnosed HIV infection can be a factor to perpetuate emergence and transmission of MDR-TB.

Despite these limitations, we have been able to study the trends of different drug resistance patterns overtime and give a better understanding of the epidemic characteristics of TB cases in mainland China. Our study identified 3 major concerns regarding TB in China. First, the MDR TB rate remains high during our study period. Second, primary transmission of MDR strains of *M. tuberculosis* is ongoing. Third, RMR strains are arising and increasing among non-HIV infected TB patients in China.

In conclusion, we demonstrated a relatively stable high level of MDR-TB in China. As one of the high MDR-TB burden countries in the world, we are facing huge challenges to meet the target of World Health Organization's new end TB strategy which aims for the elimination of TB as a public health threat by 2035.^38^ Our findings implicated that primary transmission of MDR strains of TB is ongoing, namely airborne infection control practices in China must be reinforced instantly. Besides, a commitment to DOT throughout therapy for patients with RMR, cavity or bilateral disease and for patients in particular age groups must be in place. DST for both first- and second-line anti-TB drugs is urgently needed for more individualized anti-TB regimens and for the development of a robust drug resistance surveillance system in our country.
